# Predicting gene knockout effects from expression data

**DOI:** 10.1186/s12920-023-01446-6

**Published:** 2023-02-18

**Authors:** Jonathan Rosenski, Sagiv Shifman, Tommy Kaplan

**Affiliations:** 1 School of Computer Science and Engineering, The Hebrew University of Jerusalem, Jerusalem, Israel; 2 Department of Genetics, The Institute of Life Sciences, The Hebrew University of Jerusalem, Jerusalem, Israel; 3 Department of Developmental Biology and Cancer Research, Faculty of Medicine, The Hebrew University of Jerusalem, Jerusalem, Israel

**Keywords:** Gene essentiality, Computational biology, Machine learning

## Abstract

**Background:**

The study of gene essentiality, which measures the importance of a gene for cell division and survival, is used for the identification of cancer drug targets and understanding of tissue-specific manifestation of genetic conditions. In this work, we analyze essentiality and gene expression data from over 900 cancer lines from the DepMap project to create predictive models of gene essentiality.

**Methods:**

We developed machine learning algorithms to identify those genes whose essentiality levels are explained by the expression of a small set of “modifier genes”. To identify these gene sets, we developed an ensemble of statistical tests capturing linear and non-linear dependencies. We trained several regression models predicting the essentiality of each target gene, and used an automated model selection procedure to identify the optimal model and hyperparameters. Overall, we examined linear models, gradient boosted trees, Gaussian process regression models, and deep learning networks.

**Results:**

We identified nearly 3000 genes for which we accurately predict essentiality using gene expression data of a small set of modifier genes. We show that both in the number of genes we successfully make predictions for, as well as in the prediction accuracy, our model outperforms current state-of-the-art works.

**Conclusions:**

Our modeling framework avoids overfitting by identifying the small set of modifier genes, which are of clinical and genetic importance, and ignores the expression of noisy and irrelevant genes. Doing so improves the accuracy of essentiality prediction in various conditions and provides interpretable models. Overall, we present an accurate computational approach, as well as interpretable modeling of essentiality in a wide range of cellular conditions, thus contributing to a better understanding of the molecular mechanisms that govern tissue-specific effects of genetic disease and cancer.

**Supplementary Information:**

The online version contains supplementary material available at 10.1186/s12920-023-01446-6.

## Background

Gene essentiality refers to the importance of a gene for survival and proliferation. Specifically, it refers to the fitness consequences of knocking out a gene, using loss-of-function (LoF) screening in cancer cell lines. Using data from CRISPR-Cas9 based approaches for genome-wide LoF screening, essentiality is estimated by comparing the abundances of single-guide RNAs (sgRNAs) targeting a gene at the start of the experiment to the abundances of the same sgRNAs after a few weeks of growth [1, 2].

By studying the contexts in which a gene is essential or redundant, it is possible to find candidates for drug therapies; that is, identifying those genes essential in cancer and redundant in adjacent healthy tissue [1, 2]. Moreover, by analyzing essentiality data with RNA-seq expression data of the cell lines, we can infer genetic expression profiles that cause a gene to be essential or not, thus better understanding the molecular mechanisms underlying tissue-specific essentiality and tissue-specific manifestation of genetic disease [3]. For example, the PARP1 gene has been shown to be essential in tumors where BRCA1 or BRCA2 are lowly expressed, thus making PARP inhibitors a candidate for treating subtypes of breast and ovarian cancers [4–7]. Therefore there is a need for accurate predictions of gene essentiality using gene expression as features as well as the need to understand the mechanisms which drive the context-specific behavior. An accurate model could receive as input sequencing data from a biopsy and predict whether drug target genes are essential for this individual.

Recently, project Achilles used a large set of human cancer cell lines that represent many cancer tissues to create a catalog of essential genes [2]. As part of project Achilles, there are over 900 cell lines for which both essentiality data was measured and RNA-seq was performed. In general, this data has been instrumental in the discovery of potential drug targets for cancer as well as furthering the understanding of the interaction between genes which govern how important a gene is for cell fitness. In an initial work, 516 cell lines were published by Tsherniak et al. [2], including essentiality measurements and additional molecular features, such as expression, copy number effect, and methylation. They identified 769 genes whose essentiality scores (in one or more cell lines) are several standard deviations away from the mean. Of these, 269 genes were modeled and showed statistically significant accurate predictions [2]. Machine learning techniques used to predict essentiality were developed when Gönen et al. analyzed the results of a competition to build predictive models of gene essentiality [4]. They found that the most informative features were gene expression. The final models, however, lacked predictive power since they relied solely on 149 cell lines for training [4]. Besides predictive modeling, the Achilles project has been instrumental in identifying ZEB2 as a novel dependency of Acute myeloid leukemia (AML), as well as 352 other genes which are essential specifically in AML [8]. In addition to cancer dependencies, the essentiality data was used to create a catalog of copy-number associated gene dependencies which includes 50 genes whose essentiality is dependent on copy-number [9]. The essentiality data from DepMap has not only been used to identify cancer dependencies for drug targets and predictive modeling. Kim et al. [10] developed REVEALER, a computational iterative approach that identifies groups of genomic features that together associate with a functional activation, context-specific essentiality, or drug response profile in order to understand the mechanisms which drive essentiality. More recently, Itzhacky et al. [11] used a deep learning approach to simultaneously predict the essentiality of all genes, using transcriptomes from various cell lines. This full-blown approach suffers from a lack of interpretability, and cannot be used to define the set of modifier genes affecting condition-specific essentially, which was one of the main goals of the DepMap project, and is less accurate overall compared to the model presented here. Since its publication, multiple projects utilized the data and insights provided from the DepMap project [2, 4, 8–11]. These efforts, using open access genomic and epigenomic sequencing data, have been instrumental in characterizing gene essentiality, the contexts in which genes are essential, and the interaction with the expression of other genes.

Here, we study the latest CRISPR-Cas9 based gene knockout screens and RNA-Sequencing data from the DepMap Achilles project [12] to predict gene essentiality based on expression features. We describe these genes, on which the essentiality of a target gene is dependent on, as modifier genes [12]. These are genes whose variation in expression, as a group, determines the essentiality of some target gene. We then develop machine learning models which predict for each target gene its essentiality score, using RNA sequencing data. For statistical robustness and interpretability, the model learned for each gene is based on a small set, typically between 5 and 20, of modifier genes. The genes are selected using machine learning to include only genes whose expression is associated with the essentiality of the target gene, at any cell type. These modifier genes are then integrated, for each target gene, into several types of machine learning algorithms including linear regression, decision tree and random forest models, as well as deep neural networks, in order to predict the essentiality of each target gene. As we show, this allows us to accurately predict essentiality in additional, held-out conditions, based solely on gene expression data. These results provide a benchmark for modeling essentiality using expression data from the Achilles project and identify biologically relevant sets of modifier genes which shed light on the mechanisms underlying essentiality, with far reaching applications in human genetics studies and personalized medicine.

## Methods

### Data

Gene essentiality and expression data were obtained from the DepMap project (https://depmap.org/portal/download/all/). Only cell lines which exist in both expression and essentiality data were retained. For train/test data, we randomly selected 25% of cell lines for the test set using sci-kit learn train_test_split method (Additional file 1: Table S1). For cross validation, we used five folds where each fold had cell lines selected randomly using sci-kit learn KFold method (Additional file 1: Table S2). For train/test and cross validation schemes, only train data was used for feature selection, parameter tuning, and model selection.

### Missing data removal

Cell lines with missing expression values (at modifier genes) or essentiality scores (for a target gene) were excluded from the training process.

### Feature selection

For feature selection, three scoring methods were used for each gene.

*Pearson’s correlation* We used the Scipy method pearsonr to calculate the Pearson correlation between the potential feature of gene expression and the essentiality of the target gene.

*Spearman’s correlation* We use the Scipy method spearmanr to calculate the Spearman correlation between the potential feature of gene expression and the essentiality of the target gene.

*Chi-squared statistic* For the target gene we discretized the essentiality by six quantiles: 0–0.166, 0.166–0.333, 0.333–0.5, 0.5–0.666, 0.666–0.833, 0.833–1.0. The expression of all genes was discretized using the median (either lower than or greater than or equal to, that is < or ≥). Then a contingency table was built counting the occurrence of each expression/essentiality bin value. We calculated the Chi-squared statistic of the resulting contingency table using the Scipy chisquare method.

For each gene, a score and an FDR-corrected *p*-value was calculated using the statsmodels multipletests method with parameters alpha = 0.05 and method = fdr_bh. Corrected *p*-values less than 0.05 were kept and then the top 20 genes were selected for each scoring method. The union of genes from each scoring metric were used as features [13].

**Table:** 

Feature Selection Protocol(y, gene_expression, max_num_genes = 15): let gene_expression be the expression of the set of all genes let Genes be the names of all genes let y be the target essentiality values p_vals_pearson = p-vals of pearson test with all genes’ expression and target p_vals_spearman = p-vals of spearman test with all genes’ expression and target p_vals_chisquared = p-vals of chi-squared test with genes’ expression and target selected_pearson = genes with false discovery rate below 0.05 selected_spearman = genes with false discovery rate below 0.05 selected_chi_square = genes with false discovery rate below 0.05 for each selected set of genes take the top max_num_genes selected = union(selected_pearson, selected_spearman, selected_chi_square) return selected	

The potential set of modifier genes is strictly controlled through the use of multiple hypothesis testing and only genes whose expression is significantly correlated with gene essentiality are allowed, using an FDR threshold of 5% across the entire transcriptome.

### Enrichment analysis

#### Paralog analysis

Human paralogs were defined using the TreeFam database (http://www.treefam.org/), classifying genes from different organisms to families based on homology. Paralogs were defined as any two human genes that belong to the same family and therefore are homologous. We investigated the top 100 performing genes according to the prediction versus true Pearson Correlation. For every gene, we identified the set of modifier genes and then checked whether a known paralog of the target was found in the modifier genes or not. We found that of the 83 genes in the top 100 with a known paralog, 37 had modifier gene sets that contained a known paralog. To compute a *p*-value we ran a permutation test to simulate the null hypothesis. We ran 1000 iterations where in every iteration for each of the 83 genes we used randomly selected gene sets (of the same size as the set of modifier genes identified) and counted the number of times the random set included a known paralog.

#### Pathway enrichment analysis

For the top 100 performing genes according to prediction Pearson Correlation, we tested enrichment of modifier genes by EnrichR [14] on the Kegg 2021 human pathway database [15, 16]. We used the adjusted *p*-values (Benjamini–Hochberg correction method) to determine significance. Genes whose modifier sets were enriched in at least one pathway with an adjusted *p*-value below 0.05 were considered significant.

### Machine learning models

#### Linear regression

For linear regression, expression data were first normalized to have zero mean and unit variance, using sklearn StandardScaler. Parameters of this linear transformation were fitted to training data and applied to both train and test data prior to prediction. For lasso regression, the sci-kit learn LassoCV method was used with default parameters and cv = 3. For Ordinary Least Squares linear regression, the LinearRegression method was used.

#### XGBoost

Gradient boosted trees were trained using the sklearn XGBRegressor method. The training data was split into a train and validation set (10% of train cell line IDs) with randomly selected cell line IDs being used for the validation set to optimize parameters. The learning rate was chosen as either 0.1, 0.2, 0.05 depending on the best performance on the validation set. The max_depth parameter was set to 5, n_estimators was set to 500, and early_stopping_rounds was set to 40.

#### Deep learning

Deep learning models were trained using TensorFlow, following data normalization using tensorflow.preprocessing.Normalization. Four hidden ReLU layers were used, with sizes of 50, 20, 15, and 12, respectively, and dropout parameters of 0.4, 0.2, and 0.1 for the first three layers. The final layer has output size one with a linear activation function. Every ReLU layer has an l2 regularization parameter of 0.0001. For the optimization of network weights, we used the Adam optimizer with the default learning rate of 0.001 using mean_squared_error as the loss function. For early stopping, we used tf.keras.callbacks. EarlyStopping (monitor = 'loss', patience = 100) where validation data was a randomly selected 10% of cell lines from the train data and using a maximum of 1500 epochs.

#### Gaussian process regression

For a Gaussian process regression model, a transformation was fitted so that train data would have zero mean and unit standard deviation. We used the sklearn GaussianProcessRegressor with an RBF kernel for model creation.

#### KNN

For a k-nearest neighbors regression model we used KNeighborsRegressor with n_neighbors set to 50 and weights set to be ‘distance’.

#### Decision tree

To learn decision trees we used the sklearn DecisionTreeRegressor method with max_depth set to 4.

#### Model selection

For choosing the best model the train data was split into train and validation (10% of cell lines) (note that if a specific model further splits train data to train and validation this is done with the train data defined here and not using any validation data). The model which achieved the best performance metric (root mean squared error) on the validation set was selected.

For all models, the learning stage included both parameter and hyperparameter optimization (when relevant). For this, we split the samples to held-out test data (20/25% of samples depending on whether using cross validation or not, that were not used for either optimization steps), and training samples (75%) which were further split to train (90%) and validation (10%). Parameter optimization was performed on the train set samples through the learning algorithm, and hyperparameter optimization was assessed using the validation samples. All parameters were either a fixed constant specified above in the corresponding model subsection, or were chosen as the combination of values which performed best on the validation set. If no value or list of values was specified in the methods section, then the default value of the parameter was used.

Since the feature sets output by the selection protocol might contain multiple paralog genes with similar signals, this might introduce redundancy. Here we use various model types which handle redundancy. Lasso regression is a common feature selection and feature association statistical testing framework [17, 18] that is commonly used to determine feature importance and will give lower weights to redundant features. Similarly, our deep learning models contain dropout regularizations [19] in each layer, as well as L2 regularization over weights, so again “simpler” models are preferred. Finally, tree-based models including decision trees, and gradient-boosted forests (XGBoost) are inherently sparse and regularized, and we explicitly limited the tree depth to allow for simpler models. By running various models, each with its own methods for removing redundancy, and choosing the best-performing one on a validation set, we ensure that feature redundancy is handled as efficiently as possible.

#### Performance metrics

We calculated both the root mean squared error (RMSE) and Pearson’s correlation between predicted and actuals to evaluate performance of the final models used. We used two evaluation methods. One used a random split of cell line IDs into train and test (75%/25%) and in the other, we used 5-fold cross validation. Additional file 1: Tables S1 and S2 contain the IDs used in the train/test split and 5-fold cross validation folds.

#### Algorithmic framework

Here we summarize the entire algorithmic framework with the following pseudocode which uses a random split for test/train data. The algorithm which evaluates performance using cross validation is identical except it is repeated once per cross validation fold.

**Table:** 

Computational framework overview: let g be the name of the target gene let expression be a table with all genes’ expression data let g_essent be a vector of corresponding essentiality scores of target g let cell_ids be the corresponding cell line ids # cell_ids are in the same row order as expression and g_essent train_ids, test_ids = randomSplit(cell_ids) let train_express, test_express, train_target, test_target be corresponding train/test values of expression and target essentiality scores selected_genes = Feature Selection Protocol(train_target, train_express) validation_ids, non_validation_train_ids = randomSplit(train_ids) let validation_express, train_express, validation_target, train_target be corresponding train/test values of expression and target essentiality scores let model_selected be the best performing model/hyperparameters on validation_target/validation_express model_selected.fit(train_express, train_target) model_selected.evaluate_performance(test_express, test_target)	

## Results

### Finding the set of modifier genes

**Fig. 1:**
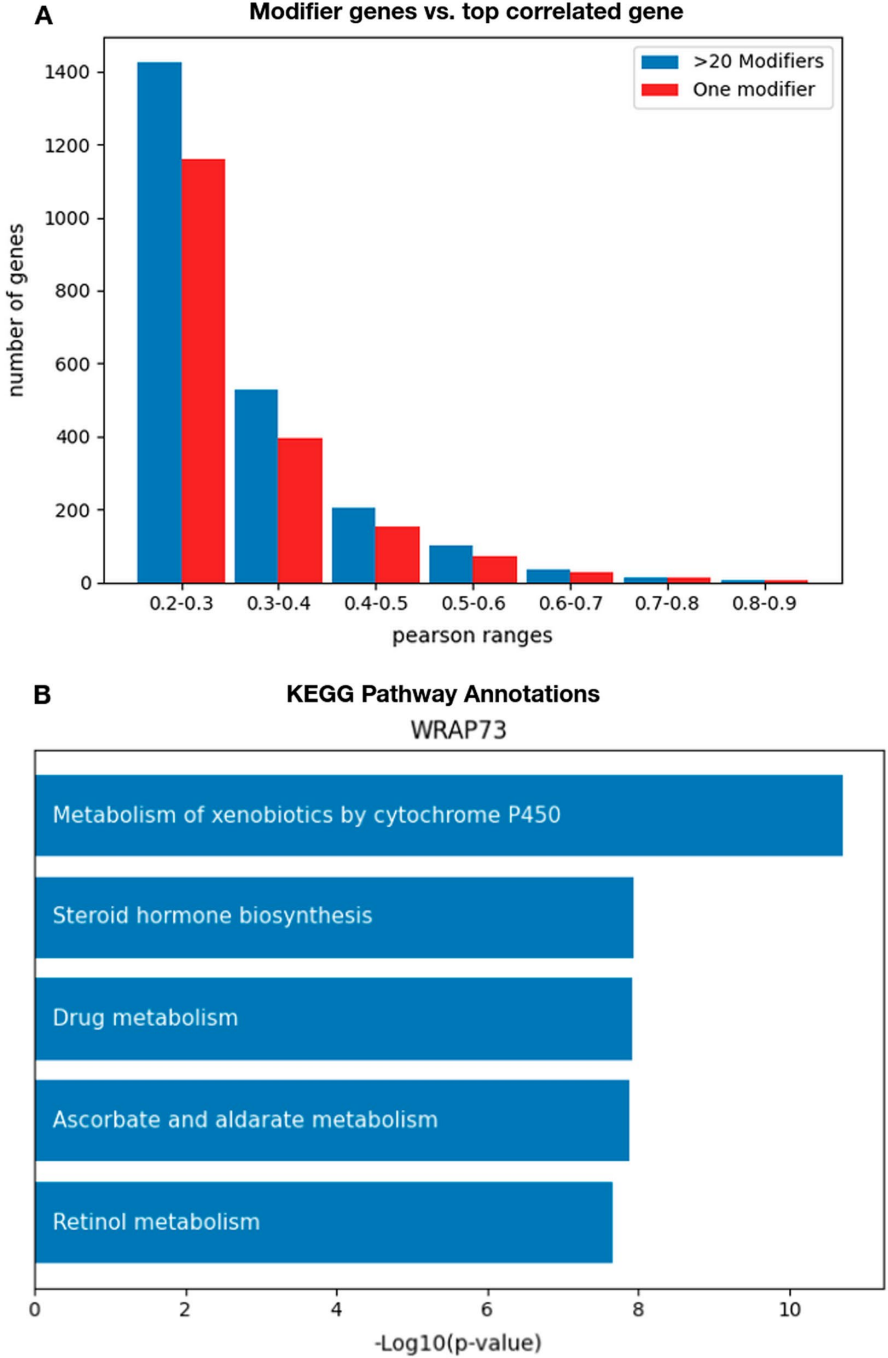
Modifier genes in models and molecular pathways. **A** A histogram of the number of target genes (Y-axis) whose predicted essentiality (X-axis) is correlated with its actual essentiality, in the test set. Blue and red bars correspond to using an unrestricted number of modifier genes (in the predictive model, blue), or when restricting the predictions to a single modifier gene (mostly correlated, red). As can be seen, single-gene models offer lower accuracy compared to larger sets of modifier genes. **B** A bar plot of − log10 transformed *p*-values of the five most significant pathway annotations in KEGG human database [16] for the modifier genes/selected feature of WRAP73 gene

Feature selection was used to identify a set of “modifier” genes whose expression is informative of the essentiality of a target gene. Previous approaches, such as those presented on the DepMap portal, have suggested using Pearson or Spearman correlation to find the single most informative gene and present graphs of correlation between the expression of the most informative gene and the essentiality of a target gene. To investigate whether there is an added benefit to using multiple modifier genes for predictive performance, we plot, in Fig. 1A, the histogram of genes that achieve different levels of predictive performance using one modifier and using the modifier genes found by our feature selection protocol.

To measure the Pearson correlation in this setup, we used a train/test split with the correlation measured on the test set. Using one modifier gene results in 1829 genes with a Pearson correlation over 0.2 while for using multiple modifiers there are 2315 such genes. Besides that, we counted for how many genes a model using one modifier beats a model unconstrained to one modifier. We use the same model type, in this case, XGBoost. Out of 9633 genes, there are 5982 genes for which using many modifier genes outperforms using just one. Overall, while using one modifier gene does provide accurate predictions for many target genes, using our feature selection protocol significantly improves predictive power.

**Fig. 2:**
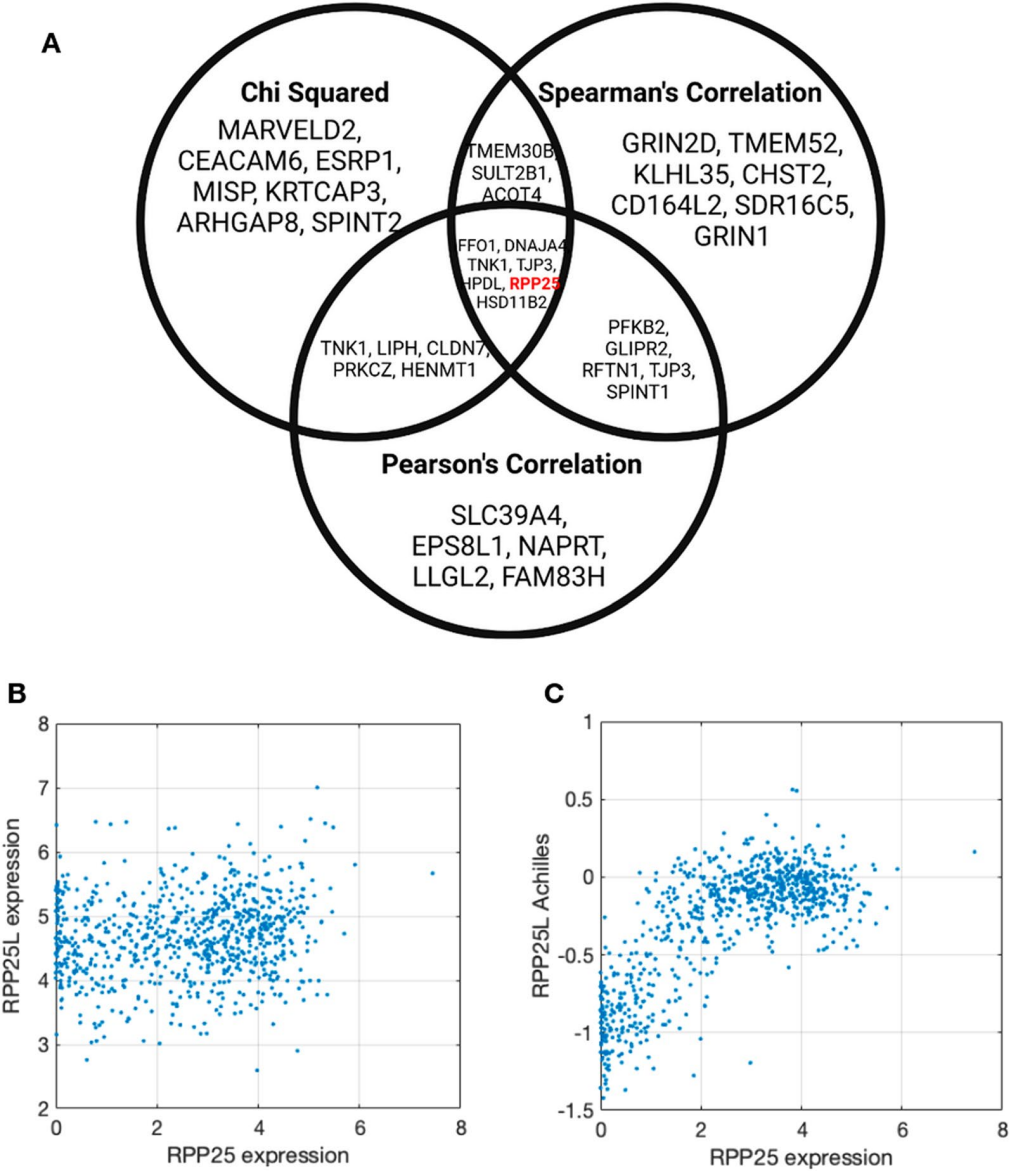
RPP25L feature selection and correlation analysis (**A**) Venn diagram of the top genes selected by association type (Chi squared, Spearman’s, and Pearson’s) for RPP25L. **B** Scatter plot showing a weak correlation between the (log_2_-transformed) gene expression levels of RPP25 (X-axis) and RPP25L (Y-axis) across cell lines. **C** Strong non-linear monotonic correlation between the expression of RPP25 (X-axis) and the essentiality Achilles score of RPP25L (Y-axis) across cell lines

Besides identifying whether there is added predictive accuracy in modeling gene essentiality using the expression of multiple modifier genes, we wanted to investigate whether the identified modifier genes are biologically related. Figure 2 shows how using our feature selection protocol for the target gene RPP25L we discover a strong interaction with RPP25, a known paralog of RPP25L. All three feature association methods find a significant relationship between the expression of RPP25 and the essentiality of RPP25L. Moreover, using XGBoost feature importance, we see that RPP25 covers over 60% of the feature importance weights, while the next feature has close to 1%. We noticed known paralogs being selected as top features for other genes as well. It is known that there are genes for which the essentiality can be explained by the expression of a paralog gene, whereby when the functionally similar gene is expressed the target gene is non-essential [3]. To investigate whether the feature selection protocol commonly identifies paralogs we counted the number of times a known paralog was identified. We did this for the 100 genes with the most predictive models, according to Pearson correlation of predictions, where a known paralog existed (total of 83 genes). For the list of known paralogs, we used the TreeFam database and considered two genes as paralogs if they are in the same family. For 37 of these 83 genes, the feature selection protocol identified known paralogs (*p*-value 0.00; see “Methods” for test details).

We also wanted to test if for these same 100 genes the modifier gene sets were enriched in common molecular pathways. We used the EnrichR tool using gget [14, 15] for enrichment analysis. Of these 100 genes, 64 had modifier gene sets identified that are enriched in KEGG human molecular pathway annotations [16] with an FDR corrected *p*-value below 0.05. We plot the *p*-values of enriched pathway gene sets for one such gene, WRAP73, in Fig. 1B.

Thus, overall, the feature selection protocol identifies sets of modifier genes whose expression is associated with gene essentiality and these genes improve predictive power. Moreover, these gene sets are biologically related in that they are both enriched in genes that are functionally similar as well as are in common molecular pathways.

### Predictive models

We use a train/test scheme and measure the Pearson correlation across predictions on the test set for each gene and calculate the corresponding *p*-value and use the Benjamini–Hochberg false discovery rate correction with a significance threshold of 0.05. Using this setup we discover 2886 genes for which we have statistically significant accurate predictions. The model type used for each gene is selected using the automatic model type selection protocol (see “Methods” for details). We also measure the 5-fold cross validation estimation of the Pearson correlation for all genes.

### Model type comparison

**Fig. 3:**
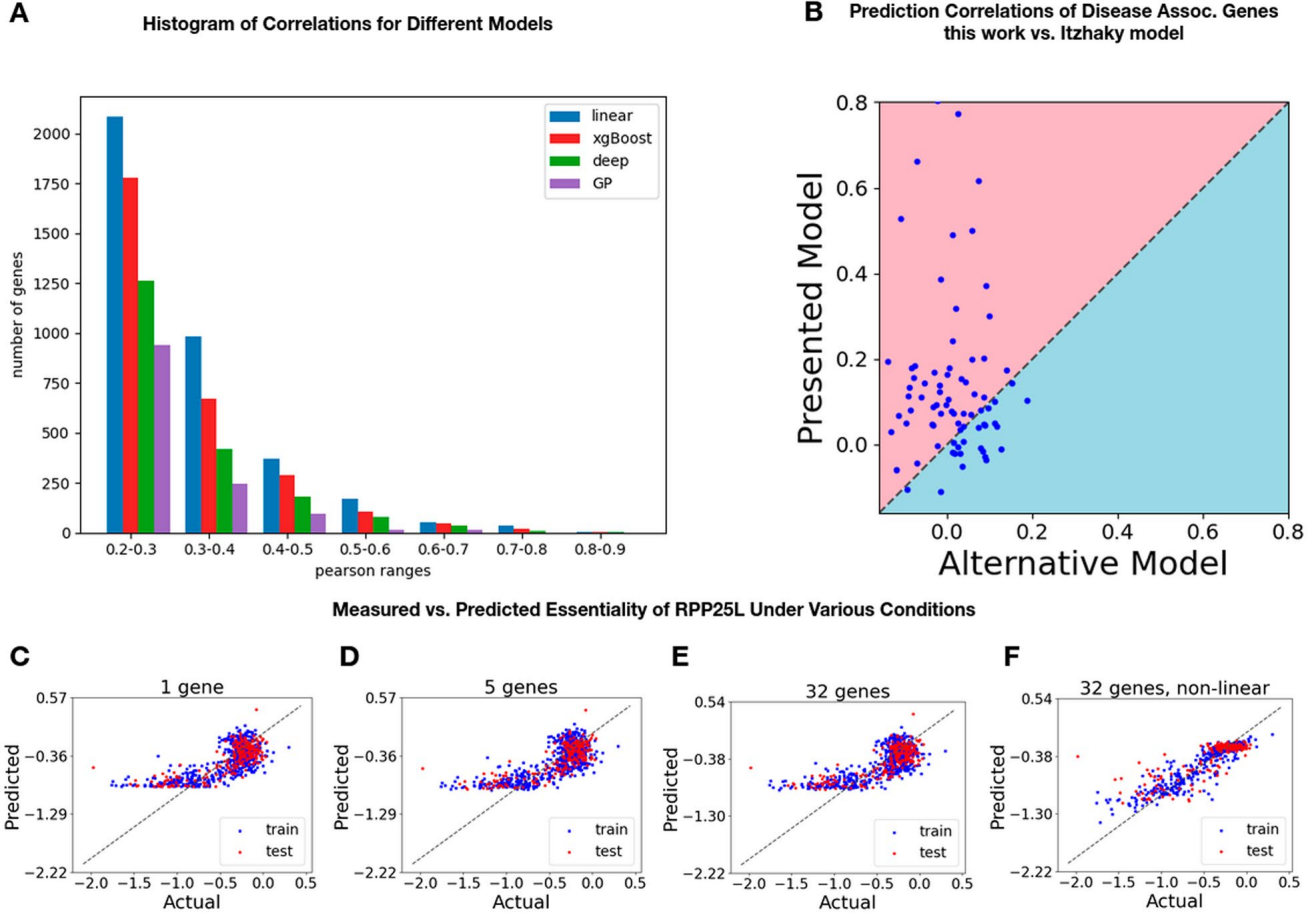
Machine learning models capture various expression-essentiality relationships. **A** Histogram of the number of genes having the specified (X-axis) Pearson correlation. Blue bars represent using a linear model, red bars use XGBoost, green bars use deep learning, and purple bars use a Gaussian Process. **B** Comparison of Pearson correlation predictions of model predictions in work presented here (Y-axis) vs. using the model of Itzhacky et al. [11] (x-axis) on a curated list of disease-associated genes ([20]. **C**–**F** Comparison of measured RPP25L essentiality score (X-axis) vs predicted (Y-axis). In all cases, data was split to train/validation set (blue), and held-out test set (red). Shown are: **C** Linear model based on the expression of RPP25 (test-set prediction r = 0.76, *p* < 2.5e−38). **D** Multiple linear regression model based on the expression of RPP25 and additional 5 covariates genes (r = 0.78, *p* < 2.8e−41). **E** Linear model using 32 covariate genes (r = 0.76, *p* < 1.7e−38). **F** Same 32 covariate genes, using a deep learning regression model combined with gradient boosting regression trees (test correlation r = 0.81, *p* < 8.5e−48)

Besides noting the overall performance after automatic model type selection, in Fig. 3A we present a histogram of the number of genes having a specific predictive Pearson score for each model type. Overall, linear models offer highly accurate predictions for most target genes, suggesting that linear associations between the expression of modifier genes and the essentiality of their targets are sufficient in most cases. We do include however other frameworks, for target genes with more complex patterns. Figure 3C–F contrasts the predicted essentiality of RPP25L using linear models with 1, 5, and 38 modifier genes (Fig. 3C–E) against the predicted essentiality achieved using a non-linear model (Fig. 3F), based on an ensemble of XGBoost and a deep learning model.

### Comparison

We compare our performance to that of the model presented in Itzhacky et al. which is the only work that predicted gene essentiality using only expression data on the recent DepMap CRISPR Cas-9 cell line data. In Itzhacky et al. [11] they use a PCA/CCA approach for dimensionality reduction, rather than a feature selection protocol such as presented in this work. While in many cases PCA/CCA approaches offer better overall performance, in this case it does not since it is done in a gene-neutral fashion. In their work they identify a transformation on the expression of all genes to be most correlated with the essentiality of all genes rather than in our work: for each gene, we find the set of genes whose expression is most correlated with the essentiality of the one target gene.

In their work, they report 1639 genes with a 5-fold cross validation Pearson correlation coefficient above 0.2 after filtering for genes with a standard deviation in essentiality above 0.1. In our work, we find 2550 such genes. Besides that, we ran both our learning algorithm and that of Itzhacky et al. on a curated list of genes that are known to exhibit tissue-specific phenotypic effects. Namely, the genes are known to have tissue-specific symptoms when there are deleterious mutations to the gene [21]. Genes such as these are likely to be essential in the tissue in which there are symptoms. The comparison, in Additional file 1: Table S5 and Fig. 3B, shows that overall the approach presented here outperforms the neural network approach of Itzhacky et al. Out of the 86 genes in the list, for 70 of them we outperform the neural network approach. The average difference between the correlation coefficient output by our model and that of Itzhacky et al. is ~ 0.117.

### Making predictions on unseen cell types

**Fig. 4:**
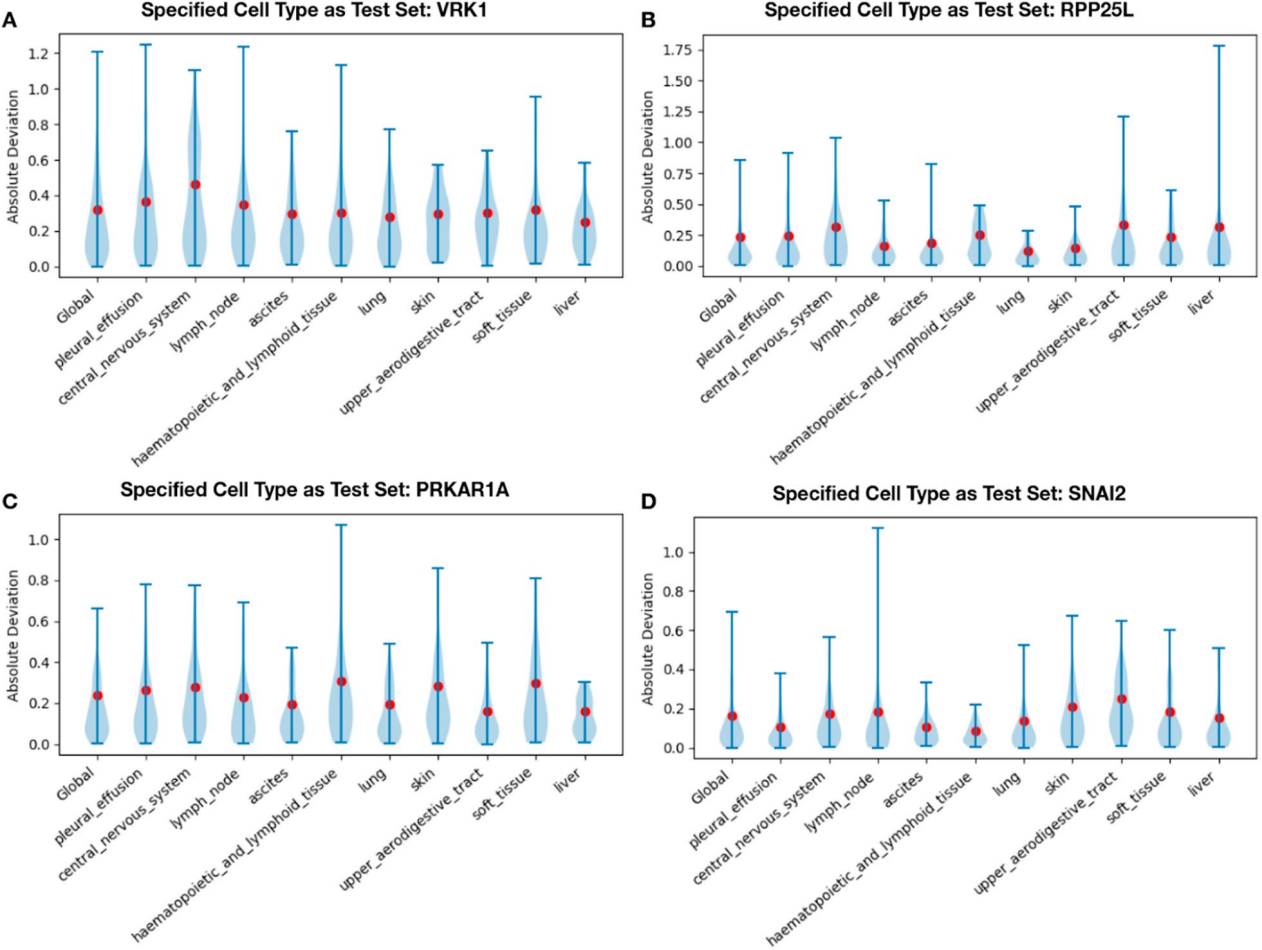
RMSE on unseen cell types in train data. **A**–**D** Violin plots displaying the distribution of absolute deviation (Y-axis) of predictions (abs(prediction − actual)) when using the specified cell type (X-axis) as held-out test data for four different genes (VRK1, RPP25L, PRKAR1A, and SNAI2). The red dots show the RMSE of the held-out cell type. The “Global” column represents having no cell-type be held out. Lower deviation suggests improvement while higher deviation indicates worse performance. The average percent difference in RMSE performance on a held out cell type compared to the random train/test split was: in **A** − 2.74%, in **B** 1.33%, in **C** − 1.25%, and in **D − **0.85%

One goal of modifier gene set models is to predict the essentiality score of a gene in novel conditions (where it cannot be directly measured—including healthy tissues). To test the viability of using our models on new unseen cell types we used an extensive cross-validation analysis, where all Achilles cell lines of a given type (e.g. “central nervous system”) were excluded from training a model for a given gene, and then the RMSE score was estimated on them. This approach is actually stricter than a simpler leave-one-out approach, where cell lines of a similar origin are included in the train and sets, which can be over-optimistic. We focused on major cell types (top 10) each containing ≥ 40 different cell lines (that were held-out), and trained XGBoost models for the disease-associated genes [21] used for comparison in the previous section. Overall, the RMSE of held-out cell lines was within 0.3% of the value obtained when training on all cell lines, on average. Interestingly, RMSE on held-out cell types was not significantly greater than control (using training samples of similar types; t-test *p*-value ≤ 0.755). This further supports the applicability of our approach to new cell types, not present in the training set data. These results are now shown in Fig. 4 and described in the main text. We present the RMSE of held out cell lines in Fig. 4 for several genes.

## Discussion

In this work, we analyze CRISPR-Cas9 essentiality screens from DepMap’s project Achilles. We introduce a feature selection protocol to identify modifier genes and use various model types to predict essentiality from expression data. Our feature selection protocol is simple and easily interpretable, which is useful for identifying sets of genes whose expression affects a target gene’s essentiality. While we do not consider transformations such as PCA, CCA, an auto-encoder, or others that might optimize predictive power, albeit obfuscating gene expression contributions, this is a direction that we think is useful for improving performance and should be considered in future work.

One standard approach to feature selection is to use linear regression with Lasso regularization and setting the regularization parameter via hyperparameter optimization techniques. Lasso regularization tends to give zero weight to non-essential features, thus it can be a popular feature selection method that is not greedy and investigates all features as a group, rather than each one’s performance. While this method is appealing, we saw much worse performance results using this method since many of the relationships between gene expression and a target’s essentiality are non-linear.

We compare predictive power over several different kinds of models and with one other work. This work is the only model known to have been developed to predict the essentiality of CRISPR-Cas9 screens. We show significant improvement over this model. This improvement seems to stem from the fact that we select features and build one model for every gene individually. Their approach seems to suffer from the fact that for many genes the essentiality scores are very noisy, or are only noise. Thus any approach which tries to incorporate features that are correlated with these noisy essentiality scores will perform less well. While our approach does have the advantage of better performance, the training time is significantly longer when making predictions for all genes. However, there is an advantage in faster training and prediction times for a single gene of interest or any small number of genes. In general, we believe a potential use case for modeling essentiality using expression data is to identify whether a specific gene is essential in some tumor for which we have expression data. In this case, we do not need to make predictions for all genes, but rather only for some genes of interest. Besides faster prediction time, in general, training time is usually not optimized since it is performed one time.

As mentioned, a potential use case for modeling gene essentiality using expression data is that we can use the models to make predictions in unseen contexts. For example, we can predict essentiality using expression data of tumors or perhaps on healthy tissue and predicting in which contexts or tissue types a deleterious mutation will have the greatest effect. Any tissue sample with a prediction significantly different than the mean prediction (student’s t-test corrected using FDR) will be classified as being affected by mutations. An analysis using the developmental single-cell gene expression data from the Descartes atlas [22], demonstrates how our previously trained models correctly predict the affected developmental tissues for several genes, including ABHD5 (associated with Chanarin-Dorfman syndrome, and predicted to affect the stomach and intestine), CACNA1S (predicted to affect muscles), FBN1 (Fibrillin-1, developing heart), FGFR2 (developing lungs), ITM2B (Cerebrum), TNNI3 (Troponin, in the developing heart). We believe this direction is promising and as more data are collected on gene essentiality and methods are developed, we will be able to accurately predict the phenotypes of deleterious mutations for many genes.

Besides using machine learning models to make predictions in clinical settings, modeling gene essentiality uncovers insights into the mechanisms of interaction between genes. We showed that the modifier genes identified commonly contain functionally similar genes as well as genes in common molecular pathways. We can also use machine learning techniques to understand the contexts in which these interactions hold. It is known that in different cell types, a gene might be involved in different pathways [23], and therefore, the essentiality will be different and the genes which affect the essentiality will be different. We use a simple linear regression model in order to visualize the effect that the expression of each modifier gene has on the essentiality of the target segregated by tissue type so that we can visualize the different interactions.

**Fig. 5:**
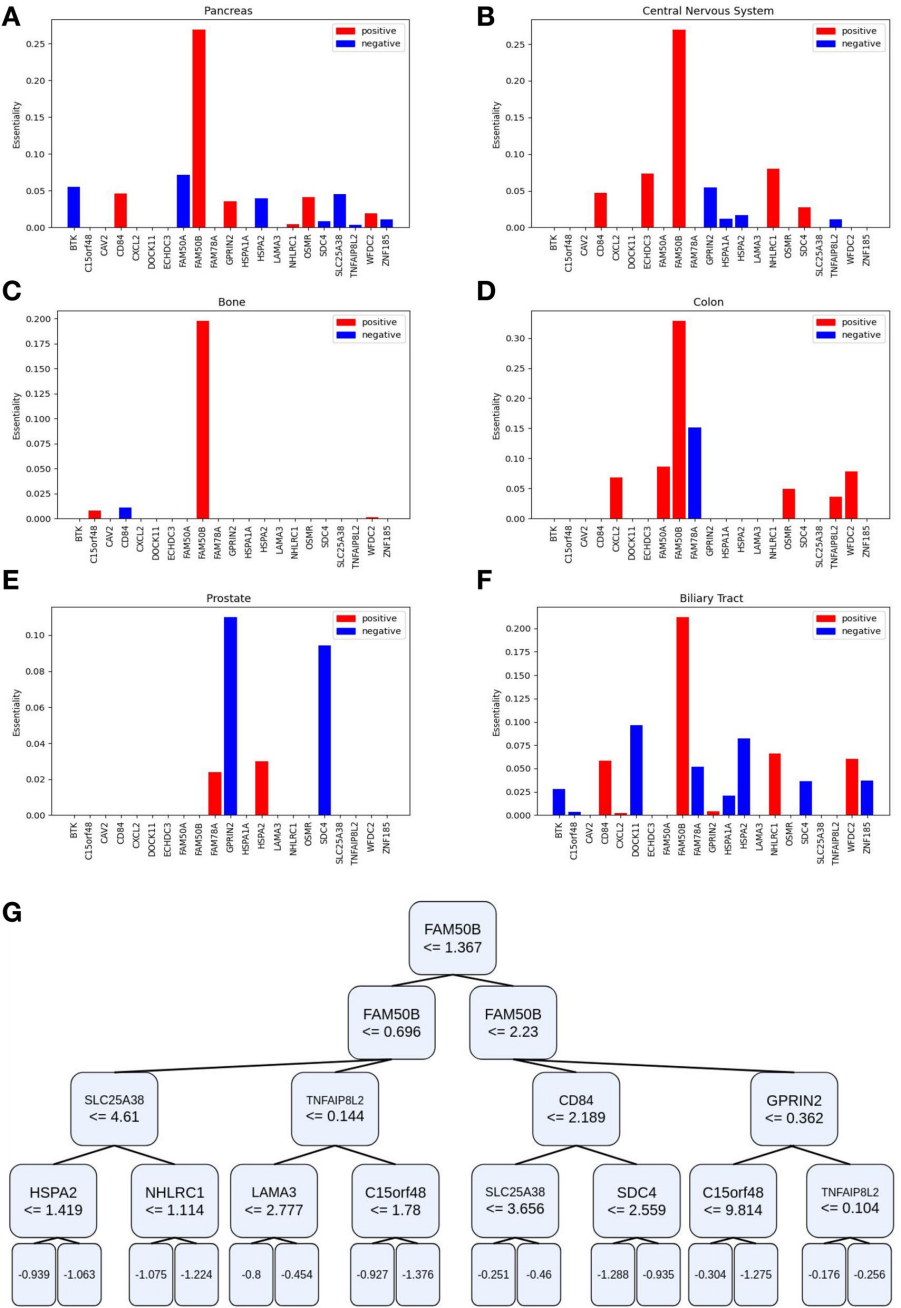
Interpretable machine learning models uncover context-dependent interactions. **A**–**F** The weights of a linear regression model predicting the essentiality of FAM50A trained on cell lines from the specified tissues. Red bars represent positive weights while blue bars represent negative weights. **G** The features of a decision tree learned using the sci-kit learn package for predicting FAM50A with a maximum depth 4. The decision tree predictions work by traversing the tree either left or right depending on whether the expression of the gene in the node is less than or equal to the value in the node (left) or not (right). The leaves represent predictions, or the average essentiality of FAM50A in the train data for cell lines that satisfy the conditions of reaching that leaf node

The top feature for predicting the essentiality of FAM50A is the expression of FAM50B, a known paralog, yet it is not the only informative feature. In Fig. 5A–F we plot the weights of features (expression of modifier genes) in red (for positive weights) and blue (for negative weights) and learn one linear regression model for every tissue type. Using the linear regression weights we can map associations and the direction of the effect segregated by tissue type. While in most tissue types FAM50B is the strongest feature, there are other tissue types where this is not the case, for example in the prostate samples. Moreover, there are various other genes that affect the essentiality of FAM50A in varying degrees of intensity.

Decision trees are a non-linear alternative to linear regression for interpretable modeling. In Fig. 5G we present the decision tree learned for predicting the essentiality of the FAM50A gene. As opposed to the case in linear regression where we segregate cell lines based on tissue type, here the decision tree segregates cell lines automatically. It begins by segregating cell lines on the most informative feature, FAM50B, numerous times. Then depending on the expression of FAM50B different modifier genes are used to determine the predicted value for the essentiality of FAM50A.

Both of these model types provide insights into the mechanisms which are associated with gene essentiality, in which contexts different genes are important for predicting essentiality. This provides further methods of advancement in understanding the interactions between genes.

## Conclusion

In this work, we demonstrate the effectiveness of using greedy feature selection protocols to create informative and accurate models for essentiality with gene expression data as input. We also show that the criteria used for feature selection both improve model predictions as well as provides informative sets of modifier genes that are enriched in common molecular pathways. Besides interpretable modeling, we show that our approach performs better than the previous state of the art in global performance metrics as well as performance on a list of genes with known genetic diseases. We introduce the transfer learning problem of predicting essentiality on healthy tissues using cancer cell line data for training. Besides making predictions on healthy tissue to predict the phenotype of genetic disease, these models serve as potential methods for discovering cancer drug targets, in identifying genes that are essential in a tumor context and not in adjacent healthy cells.

## Supplementary Information

**Additional file 1**. Supplemental Data.

## Data Availability

Essentiality scores, expression data, and copy number data are available from the DepMap project (https://depmap.org/portal/download/all). Code for learning gene models and analysis is available on github: https://github.com/yonniejon/AchillesPrediction.
